# Recurrent Swelling and Microfilaremia Caused by *Dirofilaria repens* Infection after Travel to India

**DOI:** 10.3201/eid2706.210592

**Published:** 2021-06

**Authors:** Lena Huebl, Dennis Tappe, Manfred Giese, Sandra Mempel, Egbert Tannich, Benno Kreuels, Michael Ramharter, Luzia Veletzky, Johannes Jochum

**Affiliations:** University Medical Center Hamburg-Eppendorf, Hamburg, Germany (L. Huebl, B. Kreuels, M. Ramharter, L. Veletzky, J. Jochum);; Bernhard Nocht Institute for Tropical Medicine, Hamburg (L. Huebl, D. Tappe, E. Tannich, B. Kreuels, M. Ramharter, L. Veletzky, J. Jochum);; German Armed Forces Hospital, Hamburg (M. Giese);; Radiologische Allianz, Hamburg (S. Mempel);; German Center for Infection Research, Partner Site Hamburg-Lübeck-Borstel-Riems, Hamburg (L. Veletzky)

**Keywords:** dirofilariasis, *Dirofilaria repens*, microfilariae, periodicity, *Wolbachia*, parasites, bacteria, India, Germany

## Abstract

Human subcutaneous dirofilariasis is an emerging mosquitoborne zoonosis. A traveler returning to Germany from India experienced *Dirofilaria* infection with concomitant microfilaremia. Molecular analysis indicated *Dirofilaria repens* nematodes of an Asian genotype. Microfilaremia showed no clear periodicity. Presence of *Wolbachia* endosymbionts enabled successful treatment with doxycycline.

Dirofilariasis is a zoonotic filarial infection transmitted through the bite of mosquitoes of various species. Several species of *Dirofilaria* microfilariae, most frequently *D. repens* and *D. immitis*, can infect humans. *D. repens* nematodes cause microfilaremic infection in dogs and other carnivores, which serve as reservoirs. Because humans are aberrant hosts, larvae usually develop into immature, nonfertile worms unable to produce microfilariae (*1*). Patients often report recurrent swelling with subsequent development of subcutaneous nodules, most commonly in the periorbital region (*2*). For most cases, surgical removal and histopathologic examination of the worm leads to diagnosis (*3*). *D. repens* microfilariae circulating in peripheral blood have been detected in humans only rarely (*4*,*5*), and information on periodicity of microfilaremia in aberrant hosts is lacking. One case report describes sampling of *D. repens* microfilariae from morning to midday on a single day and detection of microfilariae in the morning (*5*). Sequencing of the parasite’s mitochondrial 12S rDNA has revealed European, African, and Asian genotypes of *D. repens* microfilariae. Successful treatment of *D. repens* infection with doxycycline, which targets the bacterial endosymbiont *Wolbachia*, has been reported (*6*). To our knowledge, *Wolbachia* bacteria have not been detected in *D. repens* microfilariae of the Asian genotype.

## The Case

In April 2020, a 38-year-old man visited the outpatient clinic for tropical medicine at the Bernhard Nocht Institute for Tropical Medicine (Hamburg, Germany) 1 week after undergoing endonasal surgery for chronic sinusitis, reporting recurrent facial swelling. Nasal congestion and putrid discharge had started during a 5-week stay in Mysore, South India, his eighth trip in 5 years to the region to attend yoga classes. Two months after returning to Germany, he underwent therapeutic endoscopic septoplasty. Postoperatively, a soft tissue swelling in the right infraorbital and temporal region and general apathy developed, unresponsive to antibacterial therapy. Over 5 weeks, a low-grade eosinophilia of 0.72 × 10^9^/L (10% of total leukocytes) increased to 0.94 × 10^9^/L (14%). The result of an in-house panfilarial IgG-detecting ELISA that used a *D. immitis* extract as antigen was positive. Liver and kidney function test and serologic test results for *Strongyloides*, *Toxocara*, *Fasciola*, *Paragonimus*, *Cysticerca*, and *Gnathostoma* were unremarkable.

**Figure 1:**
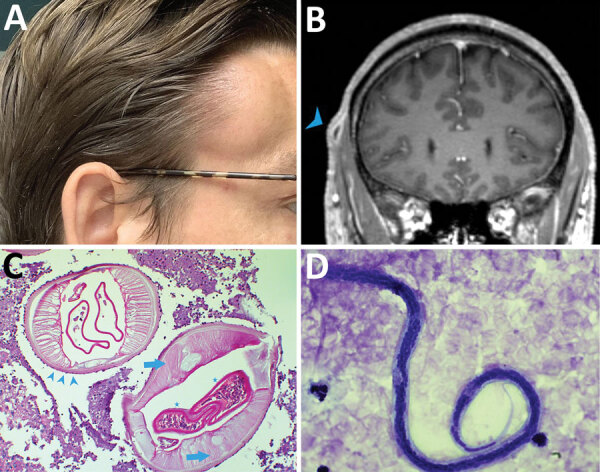
*Dirofilaria repens* infection in man in Germany after travel to India. A) Painless temporal subcutaneous swelling (image taken by the patient at the time of maximal protrusion). B) Contrast-enhanced magnetic resonance image (fat-saturated T1-weighted sequences) demonstrating a subcutaneous 10-mm lesion with central hypointensity and contrast uptake of the surrounding capsule (arrowhead). C) Cross-section through adult *D. repens* worm in subcutaneous tissue, demonstrating the cuticle with external ridges (arrow heads) and internal structures such as smooth muscle fibers (arrows) and gravid uteri (stars). Original magnification ×100; periodic acid-Schiff stain. D) *D. repens* microfilaria of the Asian genotype. Typical features include lack of a sheath, 2–3 separate nuclei in the head space, and absence of nuclei in the tip of the tail. Original magnification ×1,000 with oil; Giemsa stain.

Five weeks after his initial visit to our clinic, the patient noticed a painless temporal mass (Figure 1, panel A). Magnetic resonance imaging demonstrated a 10-mm encapsulated lenticular formation in the deep subcutaneous tissue (Figure 1, panel B). The lesion was surgically removed, and histologic examination showed an adult nematode (Figure 1, panel C). Filtration of 5 mL peripheral blood after hypotonic lysis of blood cells and subsequent Giemsa staining of the filter revealed microfilariae with the morphologic characteristics of *D. repens* (*7*) (Figure 1, panel D; Appendix; Video 1; Video 2). Sequencing and BLAST analysis (https://blast.ncbi.nlm.nih.gov/Blast.cgi) of a 463-bp fragment of the mitochondrial 12S rDNA (*8*) amplified from the adult worm and the microfilariae revealed 97.9%–99.2% homology with the Asian genotype of *D. repens* isolates from India (GenBank accession nos. GQ292761, KX265050, MT808309), followed by 95.6% homology with *D. repens* isolates from Europe (Greece, accession no. MK192091; Italy accession no., KX265072; Hungary, accession no. KX265070).

**Video 1:** Surgical removal of subcutaneous adult worm in man in Germany with *Dirofilaria repens* infection after travel to India.

**Video 2:** Microscopy of vivid *Dirofilaria repens* microfilariae in man in Germany after travel to India*.*

**Figure 2:**
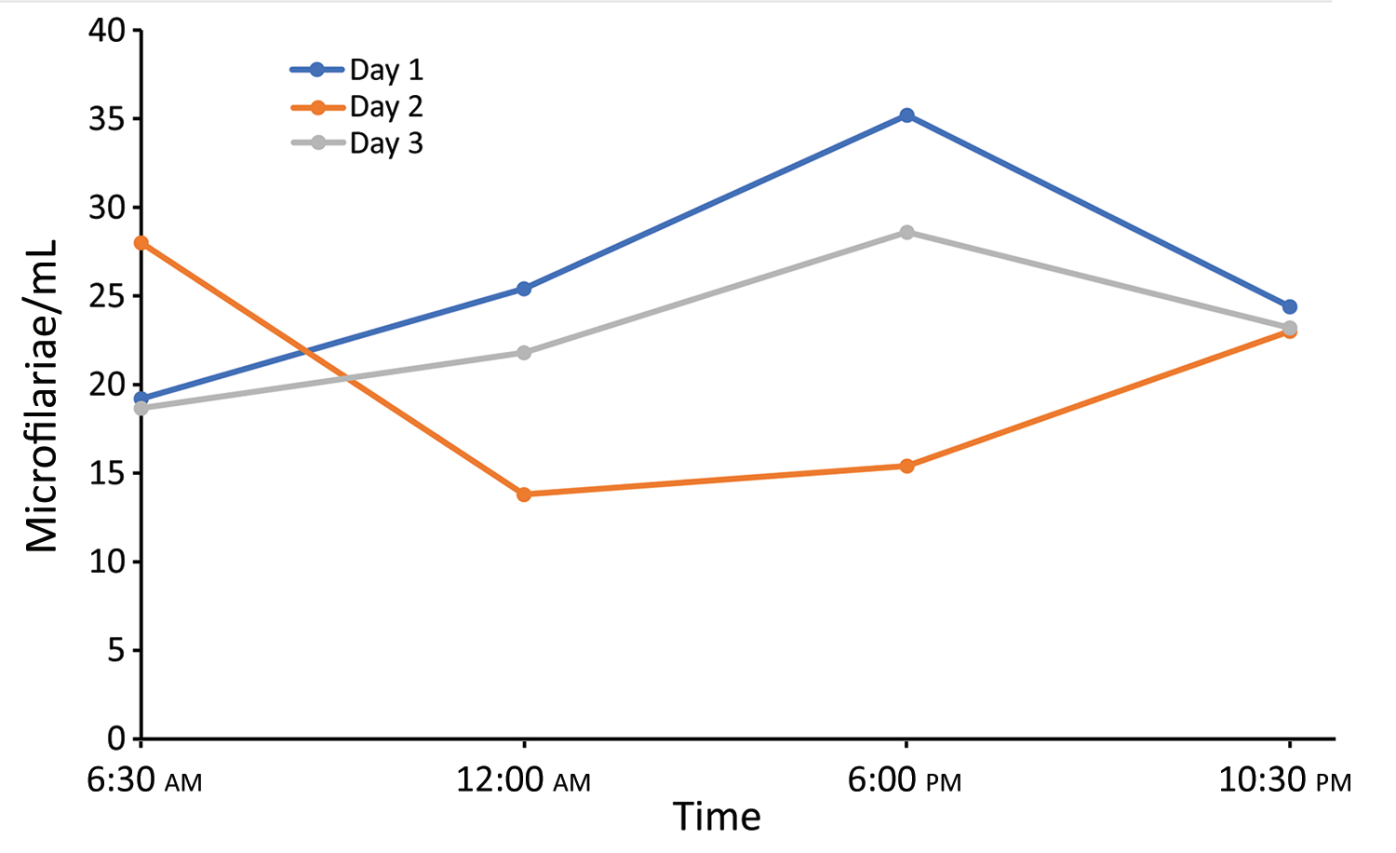
Circulating microfilariae/1 mL blood tested 4 times/day for 3 consecutive days in man in Germany with *Dirofilaria repens* infection after travel to India.

To assess possible periodicity of the microfilaremia, we sampled 5 mL of venous blood 4 times daily for 3 consecutive days and counted microfilariae after blood filtration. Blood was collected at fixed times during the day (6:30 am, 12:00 am, 6:00 pm, and 10:30 pm). Microfilariae were detectable in varying densities in all blood samples; counts fluctuated between 13 and 35 microfilariae/mL. On 2 days, the microfilaremia was highest in the evening and lowest in the morning samples, whereas on 1 day, the inverse pattern was observed. Thus, although it seems that microfilaremia substantially fluctuates during the day, this short assessment found no clear circadian rhythm of *D. repens* microfilaremia (Figure 2). To test for the presence of endosymbionts, we performed a recently published PCR that detects the FtsZ clade of *Wolbachia* (*9*). PCRs on microfilariae and adult worm samples were positive. With a goal of curative treatment, we administered doxycycline at 200 mg daily for 4 weeks, followed by a 15-mg dose of ivermectin. The patient fully recovered; eosinophil counts returned to reference ranges and microfilaremia disappeared.

## Conclusions

The areas where human subcutaneous dirofilariasis is endemic are increasing, probably because of climate change, host mobility, and global travel (*10*). Thus, cases are increasing in areas where this disease is not endemic.

We report a case of microfilaremic *D. repens* infection, which was initially noted as recurrent swelling, in a human. Molecular analysis indicated an Asian genotype of *D. repens* nematodes, which has also been referred to as Candidatus *Dirofilaria hongkongensis.* Recurrent swellings are often misdiagnosed, not taken seriously, and therefore diagnosed late. Most cases of human dirofilariasis are diagnosed after surgical removal of the adult nematode and subsequent histologic workup (*3*). *D. repens* microfilaremia in humans has been only rarely described (*4*,*5*). Several filarial species result in periodic microfilaremia (*11*), and these fluctuations can be substantial and relevant for diagnosis. Previous studies of dogs have shown that *D. immitis* and *D. repens* microfilaremia fluctuates throughout the day and peaks at night (*12*). Our results showed no clear circadian rhythm, but microfilaremia tended to be higher in the evening, similar to that of canine hosts. However, at time of blood collection, the patient had received the first doses of doxycycline, which might have affected our results.

In our investigation, the adult worm as well as the microfilariae were positive for *Wolbachia*. Doxycycline targeting this bacterial endosymbiont might thus be a treatment option similar to that for infection with other species of filariae (*13*). Molecular analysis of adult worms or microfilariae can reveal new genotypes, thereby increasing our knowledge of parasite biology and ecology (*9*). According to previous reports, *D. repens* of the Asian genotype is distributed on the Indian subcontinent (*14*,*15*). It remains unclear whether some genetic variants differ in their ability to mature and produce microfilaremia in the human host.

Localized subcutaneous swellings, particularly in the periorbital region, are a typical clinical presentation of *D. repens* infection; however, diagnosis might be difficult because of the absence of microfilaremia, eosinophilia, or positive serologic results. However, if microfilariae are detectable, they display specific features that enable microscopic differentiation. In conclusion, paramount for establishing the diagnosis of *D. repens* infection of individual patients are in-depth history taking, a high clinical suspicion, and targeted laboratory evaluation.

Appendix Images of *Dirofilaria repens* microfilariae of the Asian genotype in man in Germany after travel to India.
