# Supplementary figures and images for: Recurrent Swelling and Microfilaremia Caused by Dirofilaria repens Infection after Travel to India
Source: Emerg Infect Dis. 2021 Jun;27(6):1701–4. doi: 10.3201/eid2706.210592 (PMC8153875; doi:10.3201/eid2706.210592)

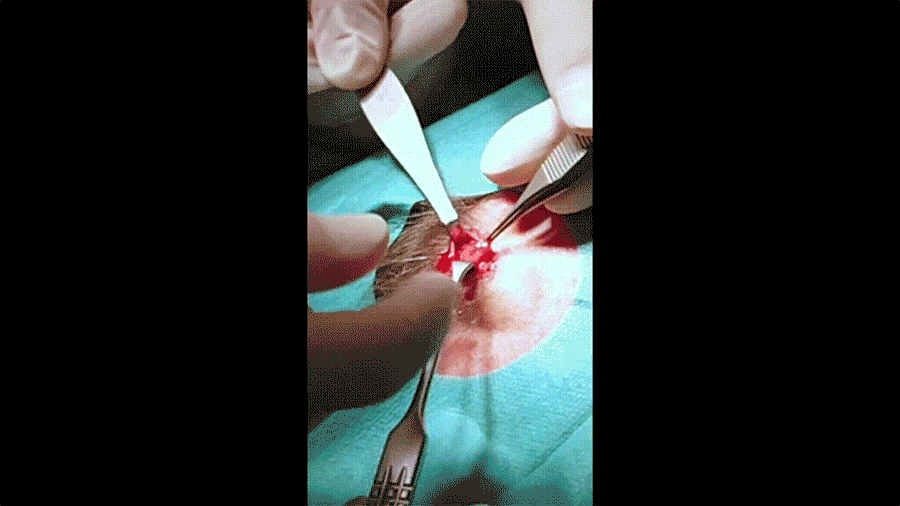

Supplement: Supplementary file 1 [file 21-0592-V1.gif]

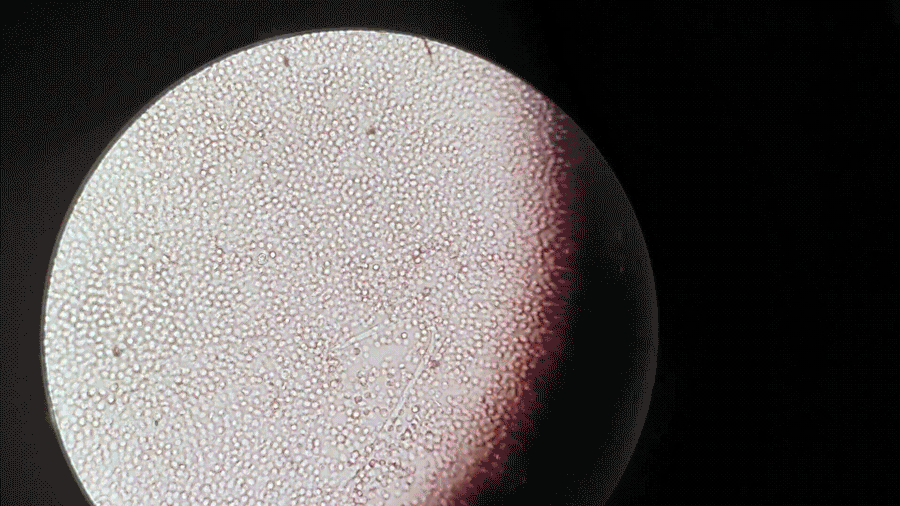

Supplement: Supplementary file 2 [file 21-0592-V2.gif]
