# Supplementary material for: Recurrent Swelling and Microfilaremia Caused by Dirofilaria repens Infection after Travel to India
Source: Emerg Infect Dis. 2021 Jun;27(6):1701–4. doi: 10.3201/eid2706.210592 (PMC8153875; doi:10.3201/eid2706.210592)
Supplement: Appendix — Images of Dirofilaria repens microfilariae of the Asian genotype in man in Germany after travel to India. [file 21-0592-Techapp-s1.pdf]

Article DOI: https://doi.org/10.3201/eid2706.210592

# Recurrent Swelling and Microfilaremia Caused by *Dirofilaria repens* Infection after Travel to India

## Appendix

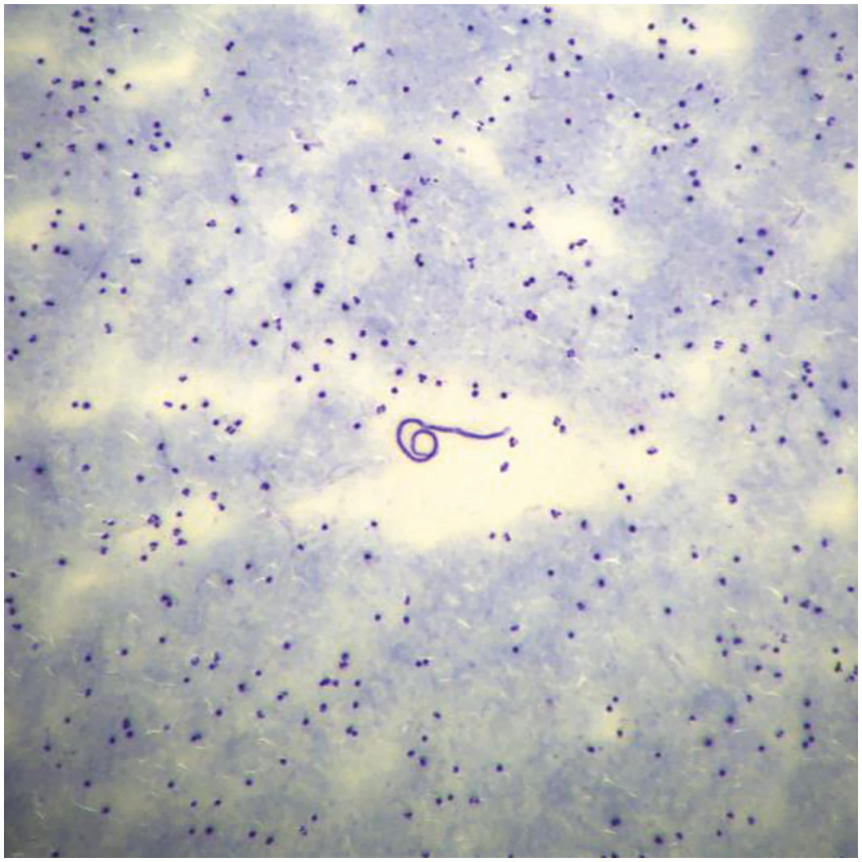

**Appendix Figures 1.** Microfilaria of *Dirofilaria repens* of the Asian genotype. Original magnification x100 with oil; Giemsa stain.

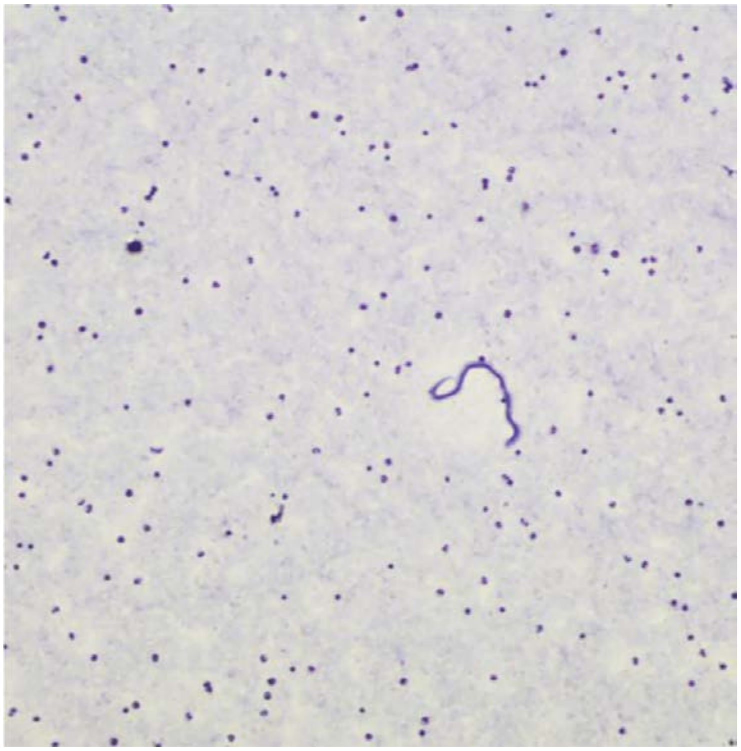

**Appendix Figures 2.** Microfilaria of *Dirofilaria repens* of the Asian genotype. Original magnification x100 with oil; Giemsa stain.

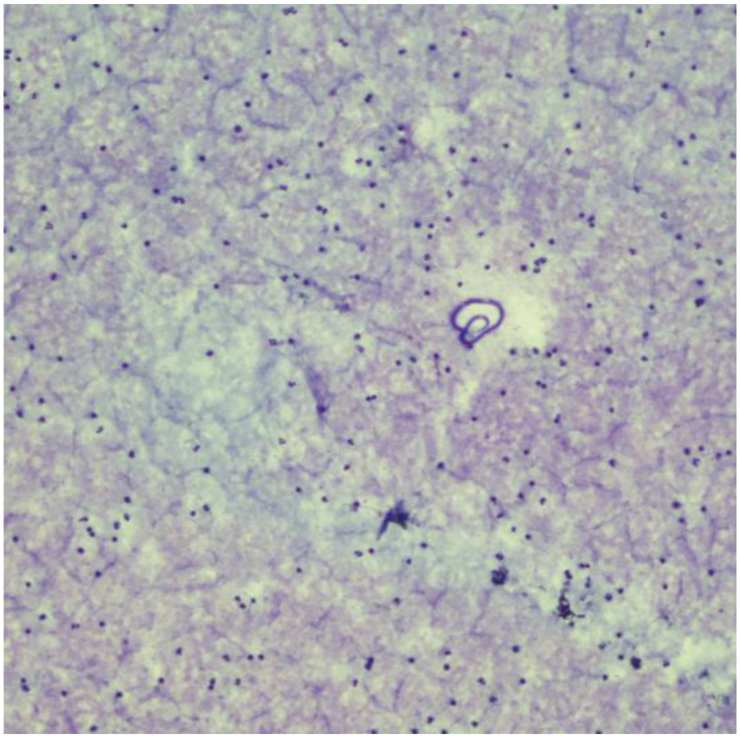

**Appendix Figures 3.** Microfilaria of *Dirofilaria repens* of the Asian genotype. Original magnification x100 with oil; Giemsa stain.

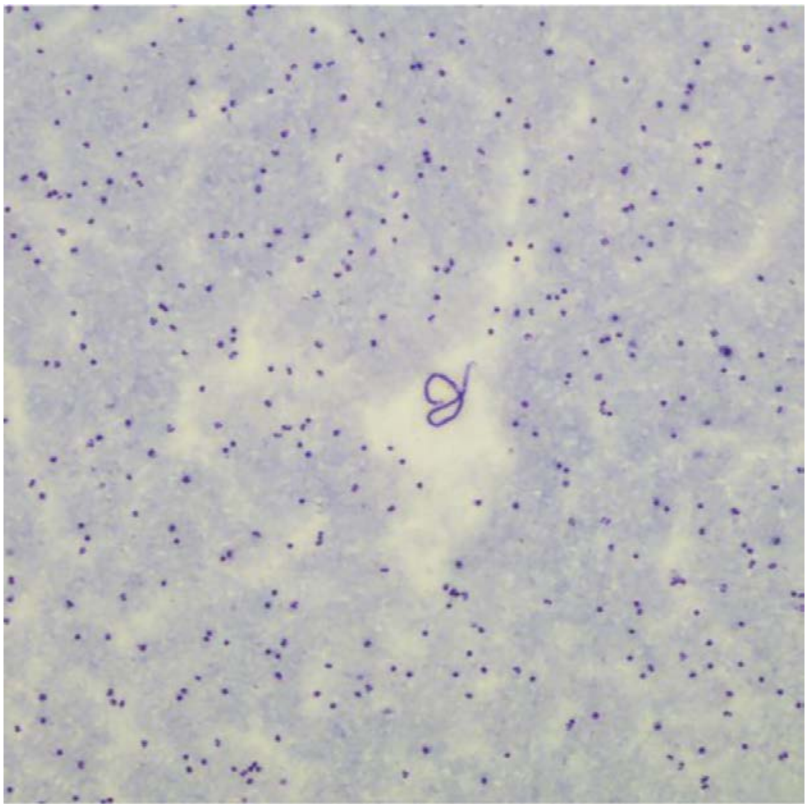

**Appendix Figures 4.** Microfilaria of *Dirofilaria repens* of the Asian genotype. Original magnification x100 with oil; Giemsa stain.

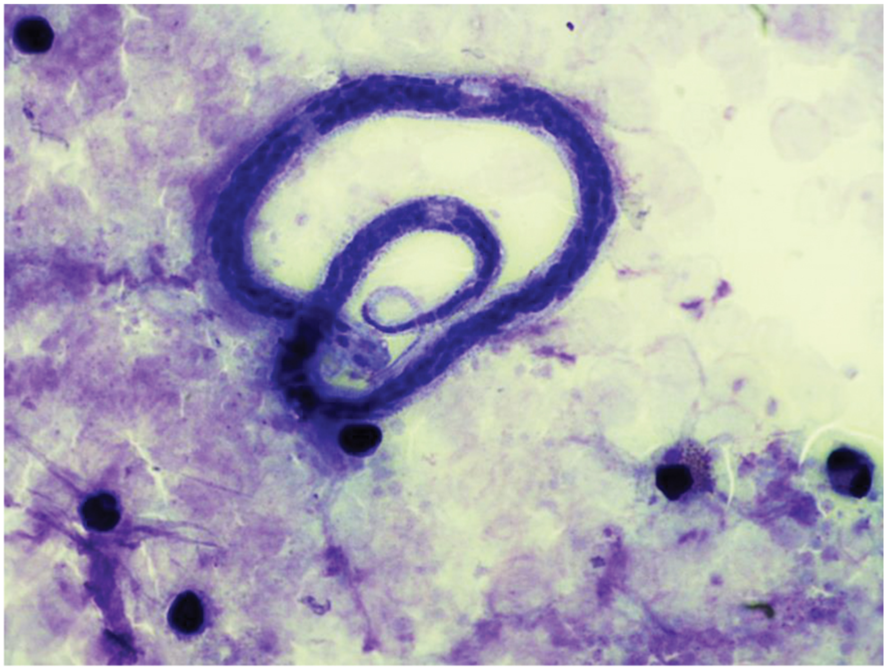

**Appendix Figures 5.** Microfilaria of *Dirofilaria repens* of the Asian genotype. Original magnification ×1,000 with oil; Giemsa stain.

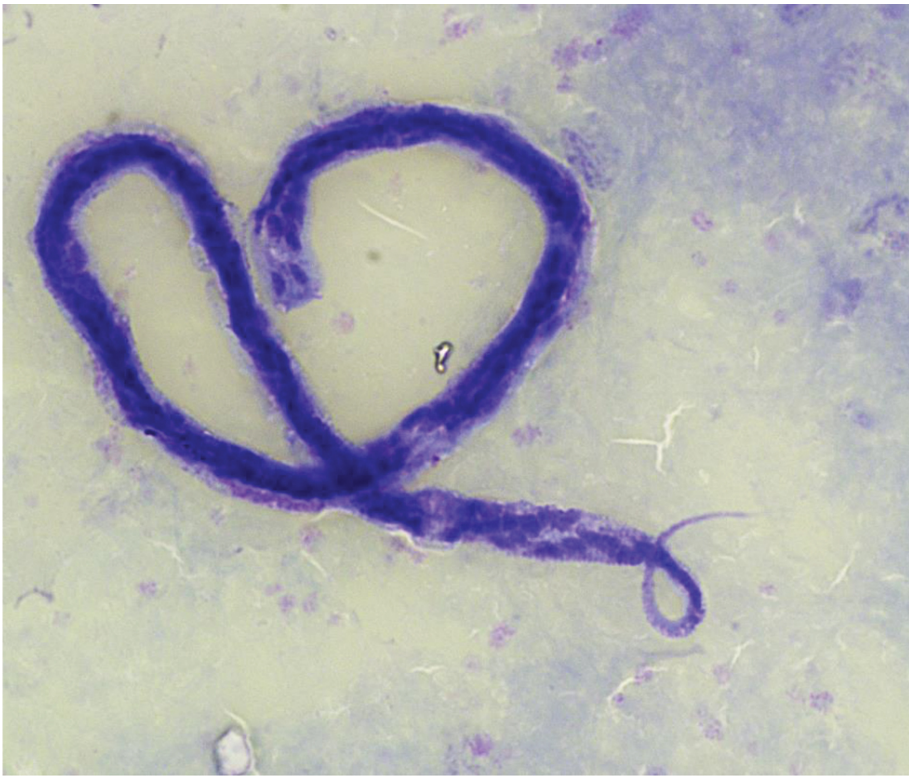

**Appendix Figures 6.** Microfilaria of *Dirofilaria repens* of the Asian genotype. Original magnification ×1,000 with oil; Giemsa stain.

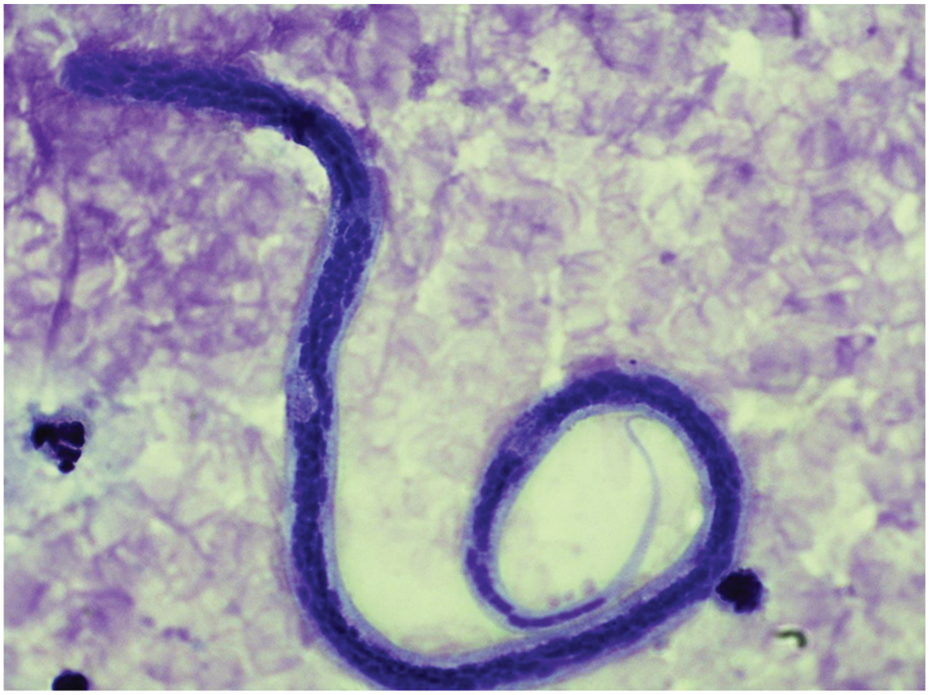

**Appendix Figures 7.** Microfilaria of *Dirofilaria repens* of the Asian genotype. Original magnification ×1,000 with oil; Giemsa stain.

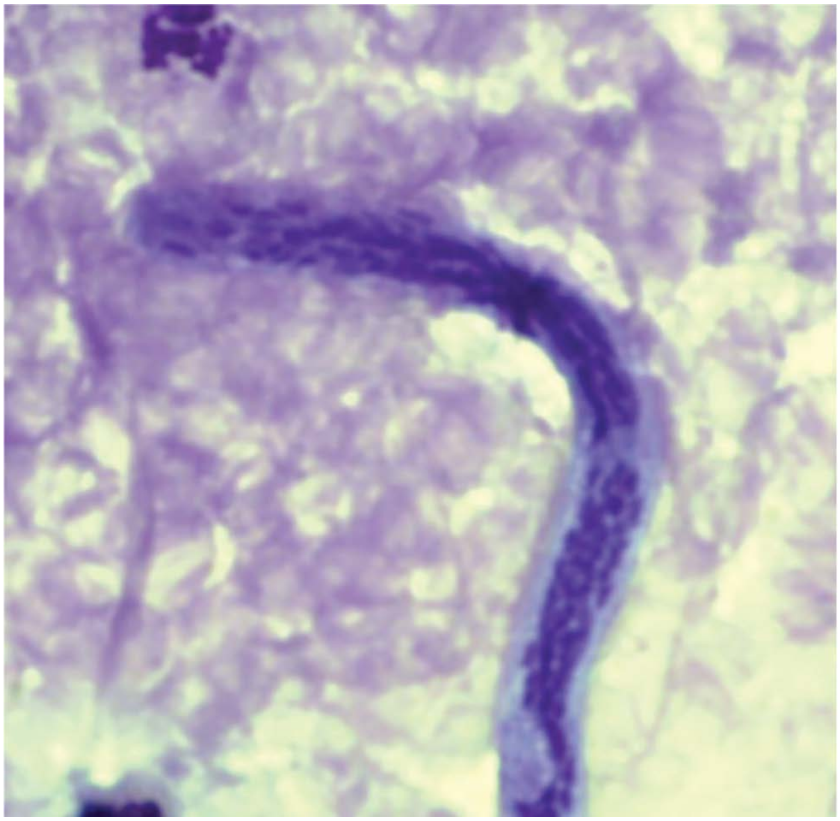

**Appendix Figures 8.** Microfilaria of *Dirofilaria repens* of the Asian genotype. Original magnification ×1,000 with oil; Giemsa stain.

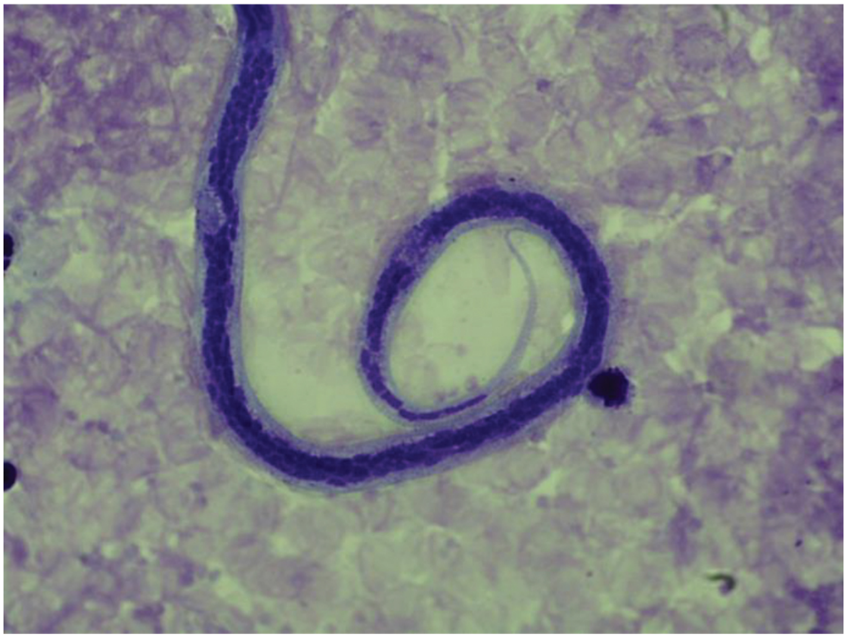

**Appendix Figures 9.** Microfilaria of *Dirofilaria repens* of the Asian genotype. Original magnification ×1,000 with oil; Giemsa stain.

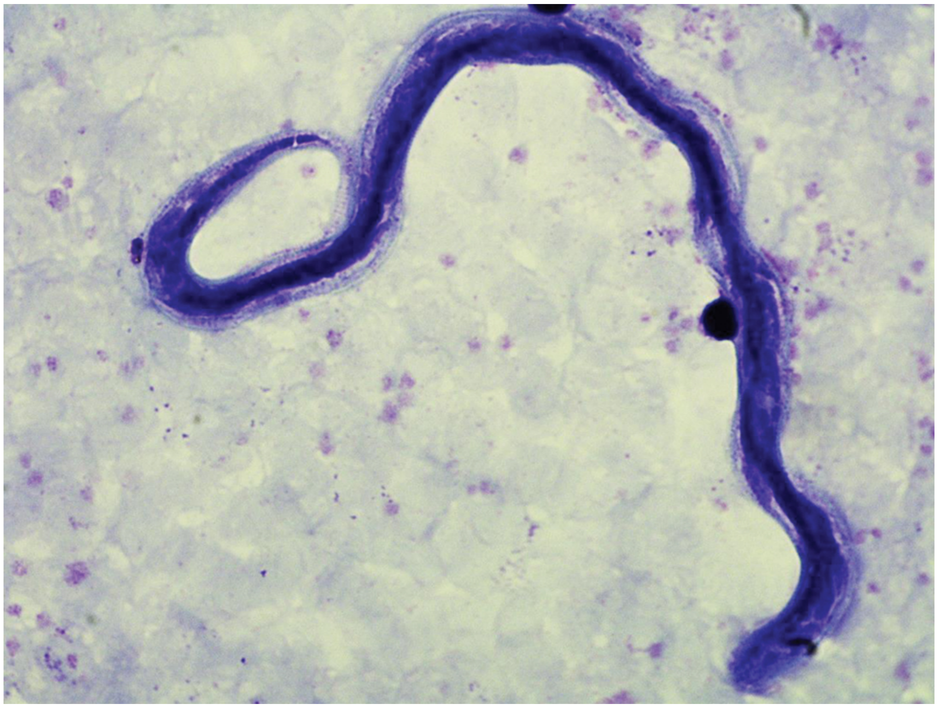

**Appendix Figures 10.** Microfilaria of *Dirofilaria repens* of the Asian genotype. Original magnification ×1,000 with oil; Giemsa stain.

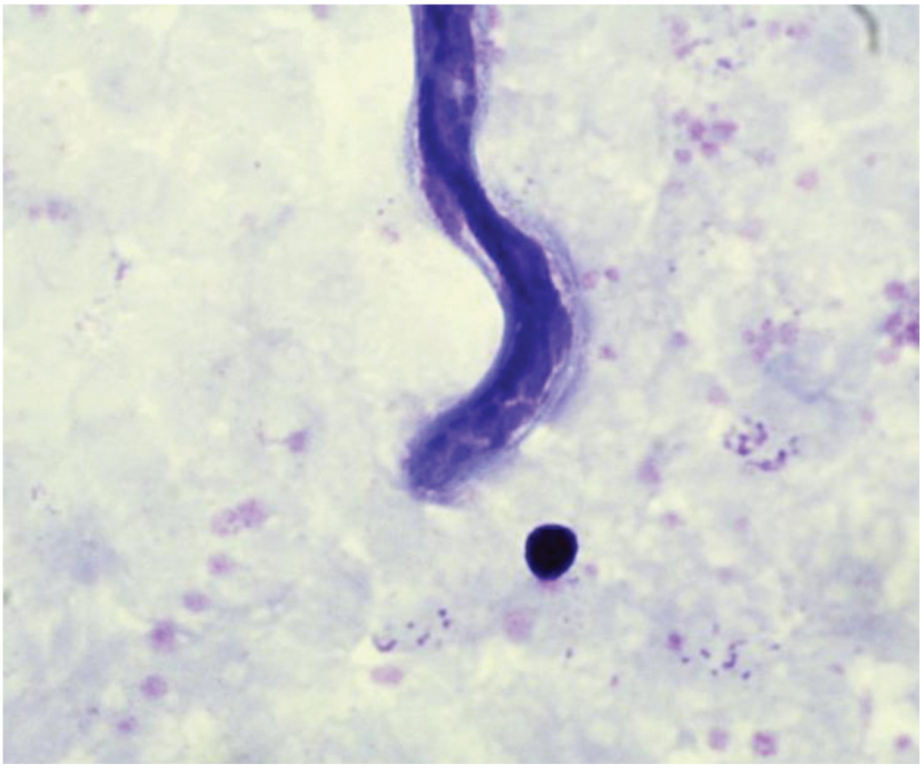

**Appendix Figures 11.** Microfilaria of *Dirofilaria repens* of the Asian genotype. Original magnification ×1,000 with oil; Giemsa stain.

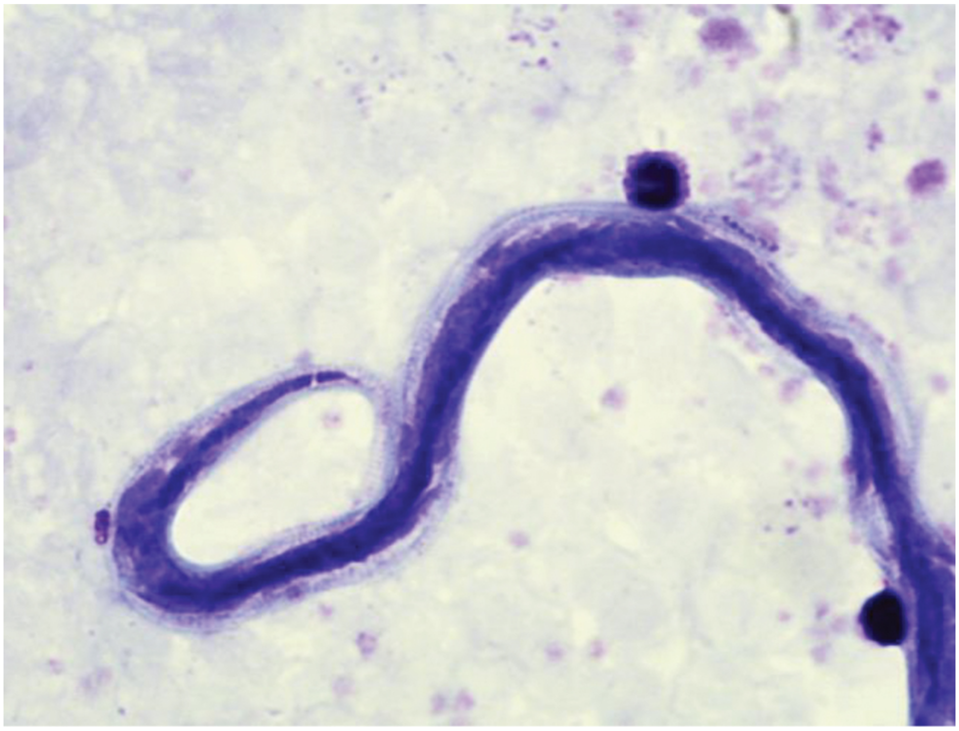

**Appendix Figures 12.** Microfilaria of *Dirofilaria repens* of the Asian genotype. Original magnification ×1,000 with oil; Giemsa stain.

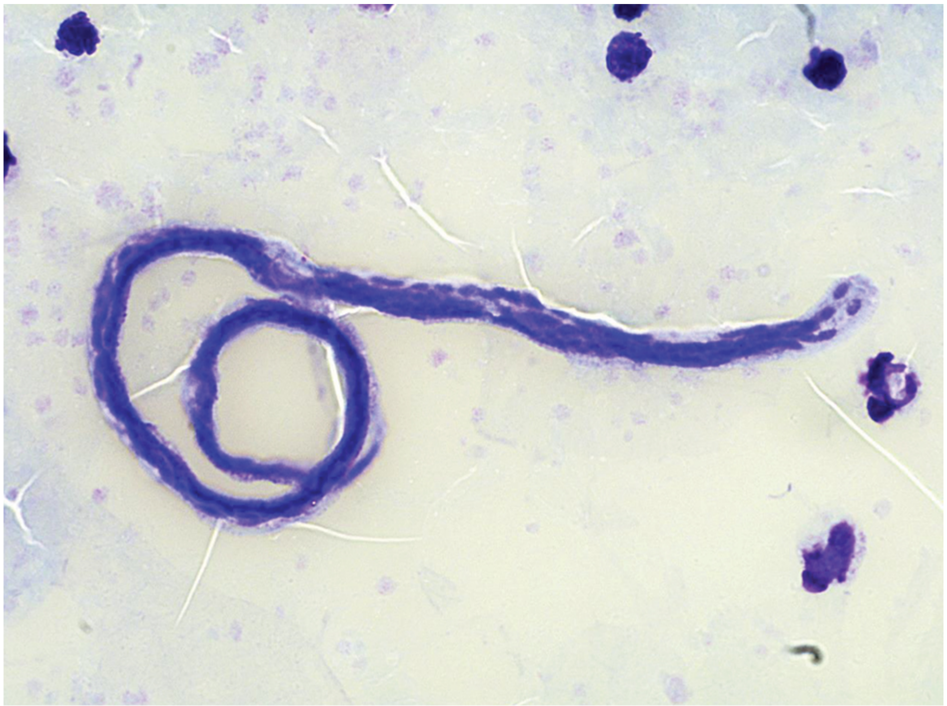

**Appendix Figures 13.** Microfilaria of *Dirofilaria repens* of the Asian genotype. Original magnification ×1,000 with oil; Giemsa stain.

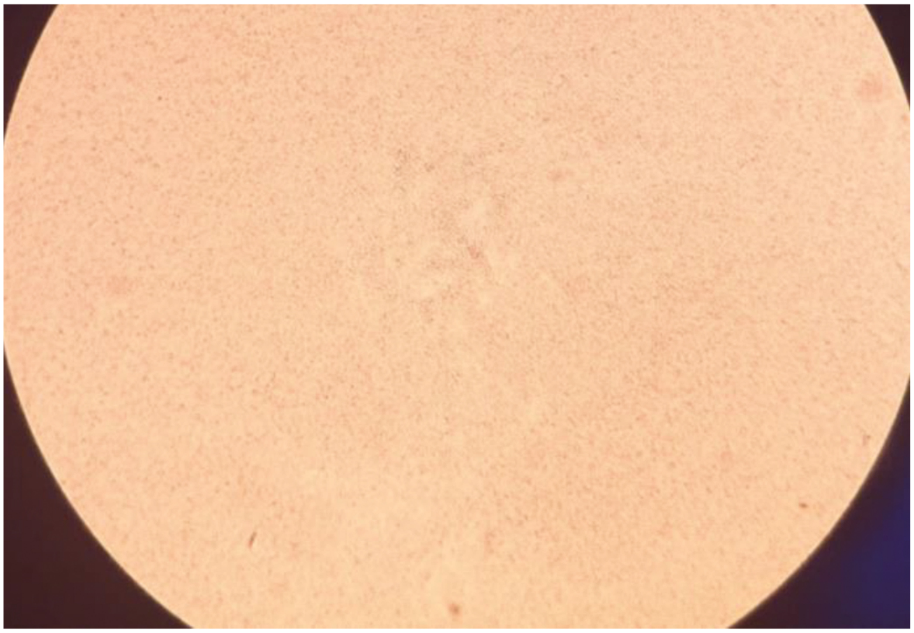

**Appendix Figure 14.** Microfilaria of *Dirofilaria repens* of the Asian genotype. Original magnification ×200 native.

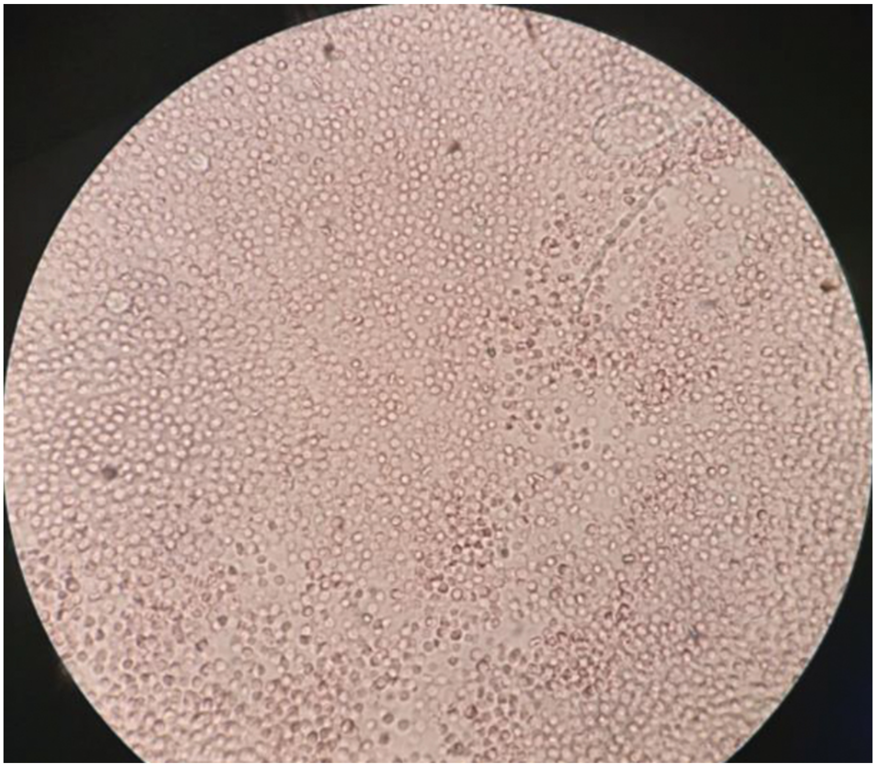

**Appendix Figures 15.** Microfilaria of *Dirofilaria repens* of the Asian genotype. Original magnification ×400 native.

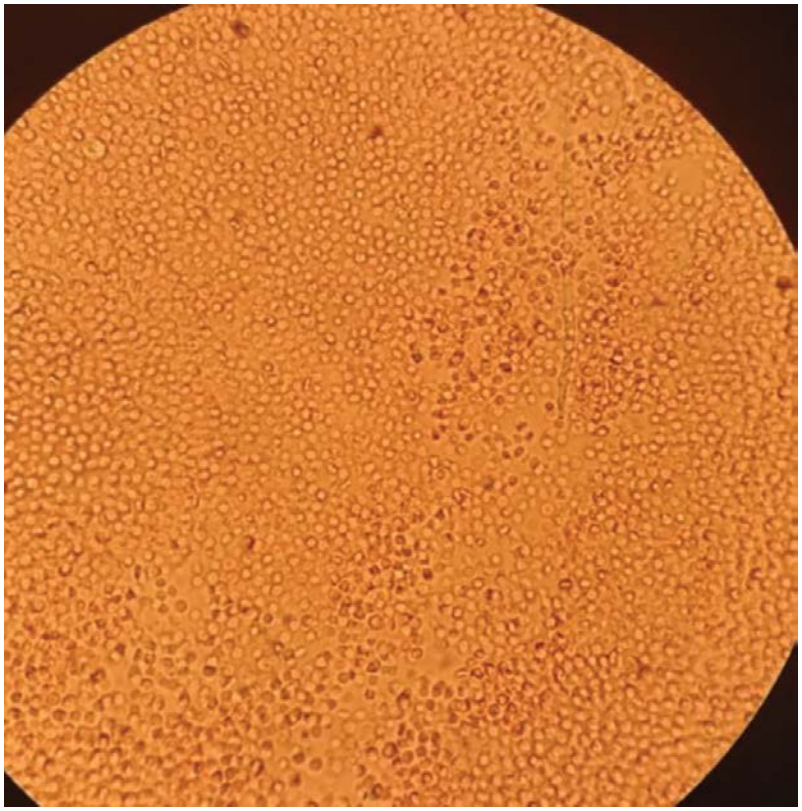

**Appendix Figures 16.** Microfilaria of *Dirofilaria repens* of the Asian genotype. Original magnification ×400 native.

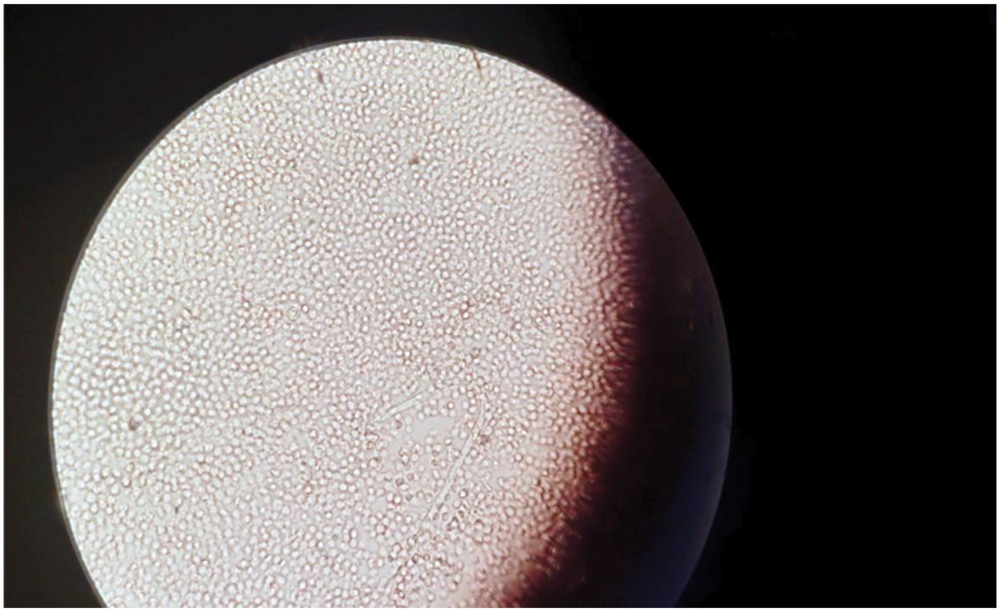

**Appendix Figures 17.** Microfilaria of *Dirofilaria repens* of the Asian genotype. Original magnification ×400 native.
